# Delayed massive subcutaneous emphysema following Robicsek closure

**DOI:** 10.1002/ccr3.2518

**Published:** 2019-11-03

**Authors:** Natalia Pavone, Federico Cammertoni, Piergiorgio Bruno, Giovanni Alfonso Chiariello, Giovanni Graziano, Massimo Massetti

**Affiliations:** ^1^ Department of Cardiovascular Sciences Cardiac Surgery Unit Fondazione Policlinico Universitario “A.Gemelli” IRCCS Catholic University of the Sacred Heart Rome Italy

**Keywords:** cardiothoracic surgery, cardiovascular disorders

## Abstract

A surgical procedure may lead to unusual and unexpected clinical scenario. Good medical practice should always keep it in mind. So, a broken sternal steel wire was the rare cause of massive emphysema.

## Graphical abstract

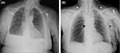

Robicsek closure is frequently used to restore sternal integrity after wound complications. We report an unusual case of massive subcutaneous emphysema following Robicsek closure that required urgent surgical revision.

A 71‐year‐old diabetic woman, with a recent history of coronary artery bypass graft using the left internal mammary artery, was re‐admitted two weeks after surgery for aseptic sternal wound dehiscence and treated using the Robicsek technique.

Postoperative chest X‐ray had shown the integrity of the Robicsek closure (Figure 1A). Several days later, she developed a massive subcutaneous emphysema of the left hemithorax extending to the left arm, bilateral supraclavicular region, and neck. Urgent chest X‐ray (Figure 1B) confirmed the subcutaneous emphysema (*). The broken steel wire of the Robicsek closure was also visible (arrow). A computed tomography scan revealed pneumomediastinum with air passing through the sternum to the left hemithorax (*)(Figure 2). The sternal bone was fractured, and the right steel wire of the Robicsek closure had tight relation with the right middle lobe (arrow) (Figure 2). Intraoperative findings confirmed a displacement of the right steel wire and a tear of the lung (Figure 3). Mediastinal adhesions had prevented air entry to the pleural cavity, leading to a pneumothorax. A sternal reconstruction was performed, and the patient was successfully discharged home. To the best of our knowledge, this is the first reported case of this complication following a Robicsek closure.

**Figure 1:**
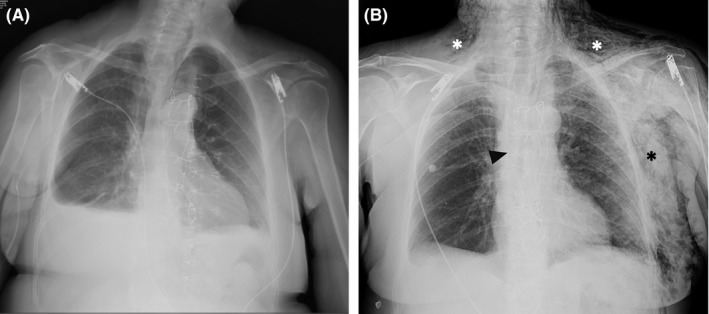
Chest X‐ray showing the predischarge integrity of the sternal steel wires (A) versus the re‐admission broken (arrow) right sternal wire causing massive subcutaneous emphysema (asterisks) (B)

**Figure 2:**
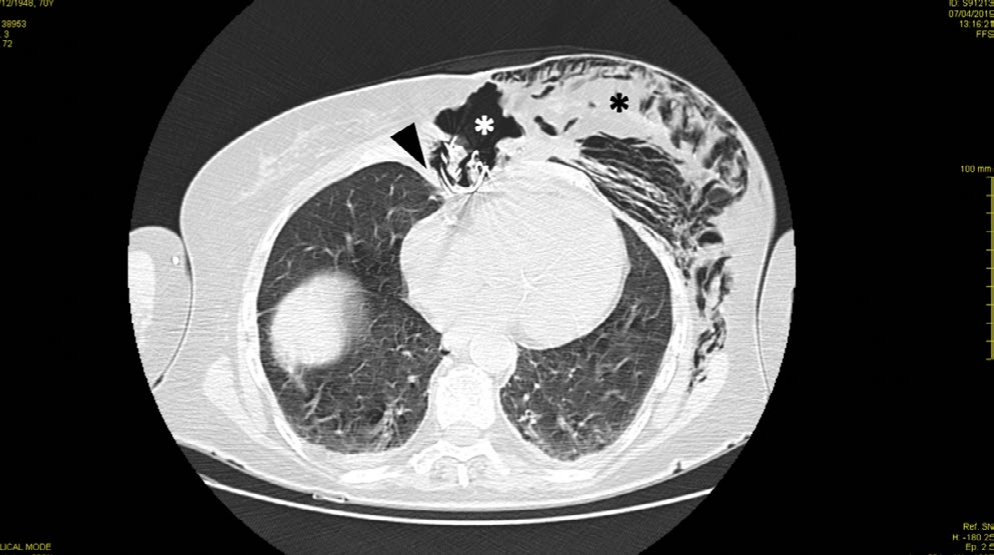
Computed tomography scan revealed pneumomediastinum with air passing through the sternum to the left hemithorax (*). The sternal bone was fractured, and the right steel wire had tight relation with the right middle lobe (arrow)

**Figure 3:**
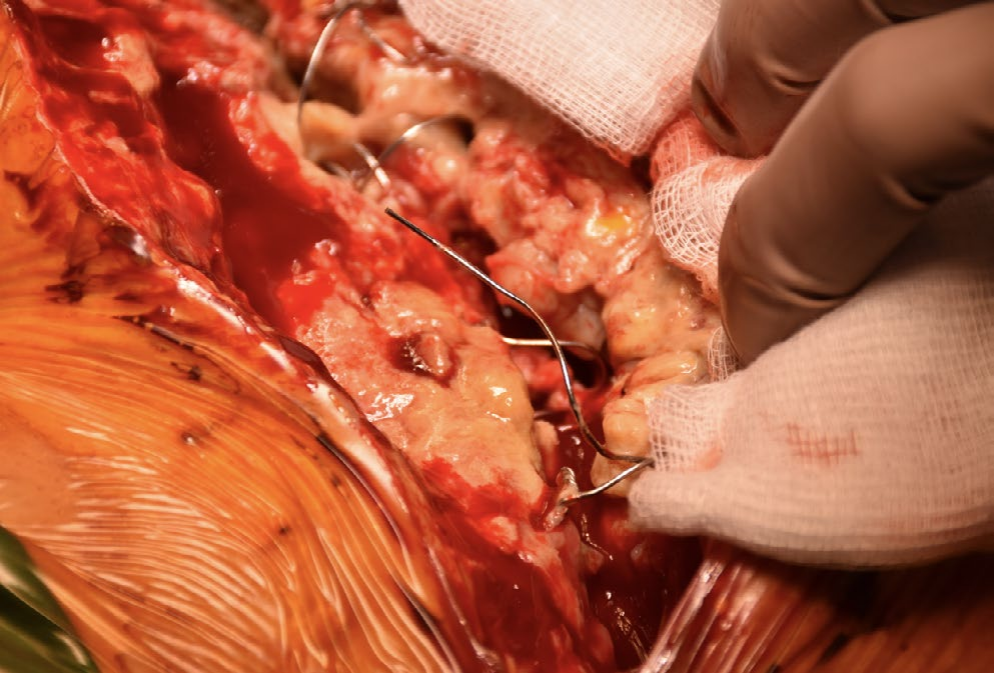
Intraoperative image showing the broken steel wire

## AUTHOR CONTRIBUTIONS

Natalia Pavone and Federico Cammertoni: wrote the paper. Massimo Massetti and Piergiorgio Bruno: supervised the paper. Giovanni Alfonso Chiariello e Giovanni Graziano: collected data and images.

